# Exploring the Impact of T2-Weighted MRI Fat Saturation on Radiomics Stability for Brain Radionecrosis Prediction After Skull-Base Proton Therapy: A Pilot Study

**DOI:** 10.3390/cancers18162636

**Published:** 2026-08-15

**Authors:** Sithin Thulasi Seetha, Giulia Fontana, Sara Imparato, Sara Lillo, Lucia Pia Ciccone, Marina Francesca Achilli, Chiara Paganelli, Silvia Molinelli, Alberto Iannalfi, Guido Baroni, Lorenzo Preda, Ester Orlandi

**Affiliations:** 1 Clinical and Scientific Department, CNAO National Center for Oncological Hadrontherapy, 27100 Pavia, Italy; 2 Radiology Unit, Clinical and Scientific Department, CNAO National Center for Oncological Hadrontherapy, 27100 Pavia, Italy; 3 Radiation Oncology Unit, Clinical and Scientific Department, CNAO National Center for Oncological Hadrontherapy, 27100 Pavia, Italy; 4 Department of Internal Medicine and Therapeutics, University of Pavia, 27100 Pavia, Italy; 5 Department of Clinical, Surgical, Diagnostic, and Pediatric Sciences, University of Pavia, 27100 Pavia, Italy; 6 Department of Electronics, Information and Bioengineering (DEIB), Politecnico di Milano, 20133 Milan, Italy; 7 Medical Physics Unit, Clinical and Scientific Department, CNAO National Center for Oncological Hadrontherapy, 27100 Pavia, Italy; 8 Bioengineering Unit, Clinical and Scientific Department, CNAO National Center for Oncological Hadrontherapy, 27100 Pavia, Italy; 9 Radiology Institute, Fondazione IRCCS Policlinico San Matteo, 27100 Pavia, Italy

**Keywords:** chordoma, radiomics, brain radionecrosis, magnetic resonance imaging, fat saturation, reproducibility

## Abstract

**Background/Objectives**: In skull-base proton therapy, T2-weighted brain magnetic resonance (MR) imaging may be acquired with or without fat saturation (FS). The present study investigated the impact of this protocol variation on radiomics feature stability and brain radionecrosis (BRN) prediction. **Methods**: Paired T2-weighted FS and non-FS follow-up MR scans of proton-treated skull-base chordoma patients (n = 52) were used to assess feature stability. For BRN prediction (CTCAEv5 grade ≥ 1), baseline planning scans of chordoma and chondrosarcoma patients (n = 80) with mixed FS protocols were used. Following automated brain tissue segmentation, 1911 radiomics features were extracted from cerebrospinal fluid, gray, and/or white matter using PyRadiomics (v3.1.0). Feature stability was quantified using Lin’s concordance correlation coefficient (CCC). Several MR image- and feature-level processing configurations were explored, and the stability of combined gray and white matter features served as the reference for selecting the optimal configuration. Features were stratified based on CCC thresholds and were subjected to univariable feature selection using a Mann–Whitney U-test. Logistic regression was used for predictive modeling and its performance was measured using micro-averaged AUC within a repeated stratified cross-validation framework. **Results**: Radiomics features were highly sensitive to FS variations (median CCC range: 0.21–0.58). Combat harmonization without image-level processing was selected as the optimal configuration, under which 30% of features achieved CCC ≥ 0.70, and 10.7% demonstrated a CCC ≥ 0.85. Restricting modeling to a highly stable subset eliminated nearly 90% of the baseline features while significantly improving predictive performance (ΔAUC = 0.05, *p* < 0.001). **Conclusions**: A subset of radiomics features robust to FS variations was identified. Use of these stable features may mitigate the impact of protocol-induced variability in mixed-FS T2-weighted MR data while simultaneously improving BRN prediction performance. These findings are preliminary and should be interpreted cautiously, given the pilot nature of the study.

## Simple Summary

Early identification of brain radionecrosis (BRN) after proton therapy is critical for skull-base tumor patients. However, variations in MR imaging techniques and data scarcity could impede the use of advanced quantitative image analysis tools such as radiomics for predictive modeling. This study investigated the impact of fat saturation (FS) on the stability of T2-weighted brain MR radiomics features in a cohort of chordoma and chondrosarcoma patients, and explored how stable features influence BRN prediction. Several image- and feature-level processing configurations were also investigated. Even with the optimal configuration, only a small fraction (10–30%) of features were stable to FS variations. Although image-level processing methods influenced feature stability, consistent improvements were observed with Combat (+55–70%). Crucially, restricting the model to a highly stable subset eliminated nearly 90% of baseline features while simultaneously improving predictive accuracy. These findings highlight the importance of selecting robust radiomics features when developing BRN prediction models utilizing T2-weighted MRI with heterogeneous FS protocols.

## 1. Introduction

Radiomics leverages advanced quantitative imaging features to develop clinical decision-support tools, in conjunction with artificial intelligence. In radiotherapy (RT), radiomics applications have been primarily focused on identifying imaging biomarkers to quantify RT effectiveness, or to facilitate early assessment of treatment-related side effects [1,2,3]. In this context, carefully selected radiomics features, combined with dosimetric parameters, can augment conventional normal tissue complication and tumor control probability (NTCP and TCP) models, presenting a great opportunity for treatment optimization [4,5,6]. For example, radiomics-integrated NTCP models could potentially improve the prediction of brain radionecrosis (BRN), a severe side effect associated with skull-base proton therapy. Nevertheless, particularly in the framework of particle therapy, for the treatment of rare diseases involving sensitive organs like the brain, the lack of data and inherent heterogeneity in imaging protocols present a major limitation in the development of robust radiomics models. This is particularly evident in magnetic resonance imaging (MRI), where although sequences such as T1-weighted (T1w) and T2-weighted (T2w) images are amongst popular modalities to detect radiation-induced BRN, the choice of the preferred sequence for investigation may vary. Specifically, T2w MRI is acquired with or without fat saturation (FS) depending on the tumor type. For example, in chordomas, a better description of the tumor extension could be achieved with FS T2w sequences while for adenoid cystic carcinoma, T2w sequences without FS are preferred [7,8,9]. Furthermore, these protocols could change over time, and across institutions, posing further challenges in gathering consistent retrospective data for radiomics analysis.

In the literature, brain MRI-based radiomics feature reliability has mostly been investigated with respect to the intra-/inter-scanner [10] and image acquisition parameter variations [11], considering the brain and its sub-regions as either homogeneous or heterogeneous targets [12]. To date, neither the impact of FS on T2w radiomics feature stability, nor its implications on the discriminative power of downstream tasks has been explored. Moreover, in MR literature, there is a lack of consensus regarding the choice of image- and feature-level processing techniques [13]. Common MR image-level processing involves bias field correction (BFC) and normalization [14,15], while Combat is one of the most popular feature-level harmonization techniques [16,17,18]. However, each of these methods involves numerous design choices that could vary depending on the settingand the scope of the analysis, while also influencing radiomics feature stability.

In this context, the primary aim of the study is to examine, in a real-world setting, how fat saturation affects the stability of radiomics features extracted from T2w MR skull-base brain tissues, and to simultaneously identify the optimal image- and feature-level processing configuration that improves stability. Additionally, we conducted a pilot study on the implications of our stability analysis findings. Specifically, we explored the predictive value of stable T2w radiomics features for BRN stratification after skull-base proton therapy. Rather than proposing a novel radiomics signature, our goal is to determine whether prioritizing stable features inadvertently excludes biologically meaningful markers while favoring reproducible technical artifacts.

## 2. Materials and Methods

### 2.1. Study Population and MRI Sequences

A total of 112 patients treated with proton therapy at our institution between 2012 and 2020 for histologically proven skull-base chordoma and chondrosarcoma were sequentially screened for eligibility. The local ethical committee approved this retrospective study (CNAO OSS 43 2021).

For the stability study, patients were excluded if the MRI follow-up protocol did not include at least one 2D T2w turbo spin echo (T2-TSE) scan with both FS and without FS (non-FS), acquired routinely during the same exam with consistent imaging protocols, or in the presence of severe imaging artifacts. Of note, follow-up exams were preferred over baseline due to increased number of consistent paired FS and non-FS T2-TSE sequences, resulting in the inclusion of 52 patients.

As for the downstream radiomics modeling study, patients with at least one T2-TSE sequence (either FS, non-FS, or both) with acceptable imaging quality in the baseline MRI exam were included. This resulted in a mixed-FS (heterogeneous) cohort of 80 patients. Of these, 35 patients had both FS and non-FS available at baseline and were evaluated separately as part of a homogeneous subgroup analysis (FS-only vs. non-FS-only). Note that 45 patients overlapped between the stability (follow-up exams) and the radiomics modeling study (baseline exams). Patient flow diagram is provided in Supplementary Materials (see Figure S1).

All MR exams were performed on a 3T scanner (Magnetom Verio/Skyra Fit, Siemens Healthineers, Erlangen, Germany) at our institution. Table 1 lists the main acquisition parameters.

### 2.2. Brain Tissue Segmentation

The most popular packages within the FMRIB Software Library (FSL v.6.0.7; e.g., FAST, BET) [19,20], Freesurfer (v7.4.0; e.g., synthstrip, skullstrip, mri_watershed) [21], and Advanced Normalization Tools (ANTs v2.5.3; e.g., antsBrainExtraction, atropos, ANTsPyNet v0.2.8) [22,23] were explored for automated skull-stripping and brain tissue extraction. The tools that achieved satisfactory brain extraction and brain tissue segmentation were qualitatively selected with the help of an experienced radiologist. Since the FS T2-TSE sequence is not part of a standard imaging protocol, automated tissue segmentation methods (e.g. FAST, ANTsPyNet) may be prone to errors, as these methods are optimized for standard, non-FS T2-TSE images. Hence, for the stability study, tissue extraction was performed on skull-stripped non-FS T2-TSE brain volumes to separate gray matter (gm), white matter (wm), and cerebrospinal fluid (csf). Subsequently, non-FS T2-TSE images and segmentations were rigidly co-registered to the tumor-localized FS T2-TSE sequence using SimpleITK (v2.3.0) [24,25], thus deriving two skull-base region focalized T2-TSE sequences. For the downstream task, since non-FS sequences were not available for all patients and to better reflect a realistic modeling workflow involving mixed-FS protocols, tissue extraction was performed independently on both sequences.

### 2.3. Image-Level Processing

MR image intensities are relative non-standardized units reflecting qualitative scanned tissue contrast. Currently, there is no consensus regarding the best way to pre-process brain MRI in radiomics studies. However, the most common steps include (i) spatial volume resampling; (ii) BFC; and finally, (iii) MRI intensity normalization [14].

Initially, we resampled all the images to a common isotropic voxel spacing of 2 × 2 × 2 [mm^3^] using 3rd-order bspline interpolation. Given the highly anisotropic original voxel spacing (see Table 1), this served as a reasonable compromise to avoid large interpolation artifacts, while ensuring rotational invariance of texture radiomics features in the subsequent feature extraction step, as recommended by IBSI. Three common N4 BFC approaches were explored by altering the region used for bias correction in ANTsPy (v0.4.2) [26]: (i) default: a binary mask is generated automatically using naïve thresholding; (ii) brain mask: the segmented brain forms the foreground; and (iii) gwm mask: treats combined gray and white matter as the foreground. Finally, MR intensity normalization was applied based on the z-score normalization using subject-wise mean and standard deviation computed on a target volume. Although the use of the entire brain or gwm as targets was more represented in the literature, we explored four variations: brain, gm, wm, and gwm. The use of gm- and wm-based normalization was inspired by approaches such as WhiteStripe [27], where normally appearing wm is utilized to yield more robust results. After standardization, to remove the impact of outliers, intensity values were clipped to lie within [−3, 3] as recommended by IBSI [28,29].

### 2.4. Radiomics Feature Extraction and Harmonization

After applying each image-level processing configuration, radiomics features were extracted using IBSI-compliant PyRadiomics (v3.1.0) open-source package. A total of 1911 radiomics features (first order, texture) were extracted from each target—gm, wm, csf, gwm, and whole brain—present in T2-TSE (FS/non-FS) sequences. Additionally, all standard image filters available in PyRadiomics were applied [30]. Bin count discretization strategy (bin number = 32) was adopted before extracting texture features [14,31]. This aligns with IBSI recommendations for arbitrary MR intensities and has been shown to improve overall MR feature stability [31,32], with 32 bins specifically identified as optimal for brain MR radiomics feature stability [14]. Finally, the NeuroCombat-sklearn (v0.2.10) package [33,34] was used to harmonize features (i.e., feature-level processing) between the two sequences [35]. Specifically, the batches were defined strictly by the fat saturation status (i.e., FS vs. non-FS), without incorporating additional biological covariates. The feature extraction settings remained identical for both the stability and downstream modeling studies. Of note, to harmonize the modeling cohort, the Combat harmonization parameters estimated from the stability study were directly applied without additional refitting.

### 2.5. Stability Analysis

A total of 40 configurations (20 image-level processing configurations, applied both with and without Combat) were investigated for each target tissue: gw, wm, csf, gwm, and the whole brain. Table S1 in the Supplementary Materials lists all these configurations. The optimal configuration was defined as the one that improved the stability of radiomics features extracted from the reference tissue, which was chosen to be gwm. By choosing the gwm region as the reference tissue, our goal was to identify a set of stable radiomic features that are broadly generalizable to downstream prediction tasks involving targets within the intracranial tissue.

Feature stability was quantified using Lin’s concordance correlation coefficient (CCC) [36] and was stratified based on its stability level, as in [11]: poor (CCC < 0.5), moderate (0.5 ≤ CCC < 0.7), good (0.7 ≤ CCC < 0.85), and excellent (CCC ≥ 0.85) [37,38,39,40]. The impact of each configuration was measured using their median CCC, with 95% confidence intervals (CIs) estimated via bootstrap resampling (n = 2000). To identify the optimal configuration, the one with the highest median CCC was compared against all others using a Wilcoxon rank-sum test with Bonferroni correction. A *p*-value < 0.05 was considered statistically significant. Finally, to prevent overfitting, we applied the principle of parsimony, i.e., we selected the simplest configuration among those with statistically comparable performance.

**Figure 1:**
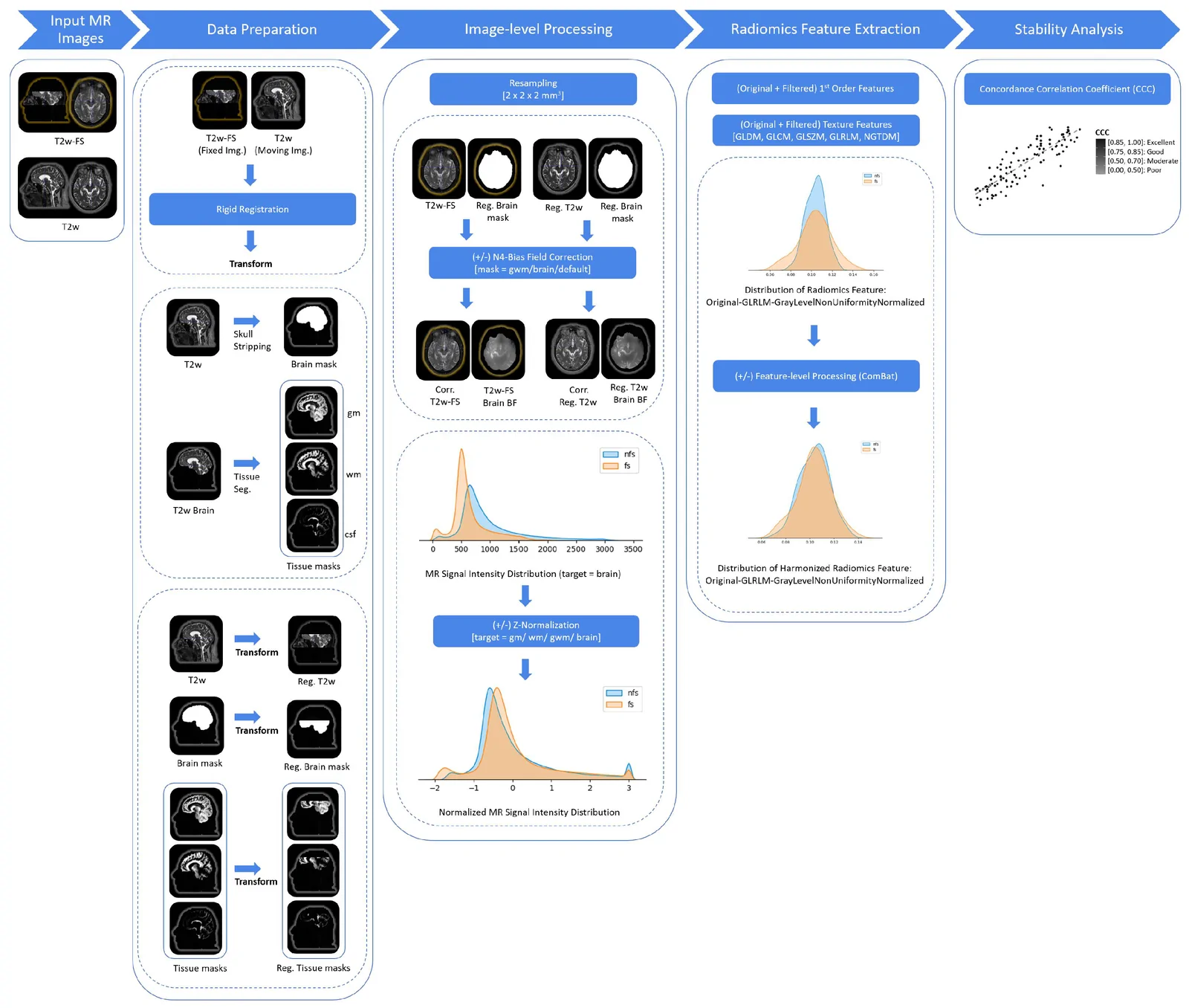
Workflow of the study, from magnetic resonance imaging acquisition to radiomics feature stability analysis.

A multivariable linear mixed-effects regression analysis was also performed to explore the influence of processing methods on the stability of reference tissue features. To account for dependencies across processing configurations, random intercepts were included for radiomics features and image filters. The baseline configuration <no BFC, no Normalization; no Combat> served as the reference level. Additionally, we investigated their impact considering subsets based on image filters and feature families. Given the retrospective nature of our study, we also explored how variations in repetition time (TR) affected the reproducibility of T2w FS and non-FS gwm features. Only TR was selected, as it was the main acquisition parameter that varied across sequences (see Table 1). Figure 1 summarizes the methodological framework associated with the stability study. The Python-based implementation of the project is available as open-source at https://github.com/sithin-cnao/t2w_stability.git (accessed on 10 August 2026).

All analyses were performed with Python (v.3.7.5) [41], while R (v4.0.1) [42] was used for graphical illustrations.

### 2.6. Radiomics Modeling

The radiomics modeling task was designed to investigate the impact of stable features extracted from baseline (planning) scans on BRN prediction. BRN was graded in accordance with Common Terminology Criteria for Adverse Events (CTCAEv5). Grade 1 or higher (G1+) BRN was considered as the primary outcome variable. This decision was driven by the potential for G1 to evolve into higher grade toxicities in the long term, coupled with a low prevalence of high-grade events (G2+) in our cohort. BRN diagnosis and grading were assessed based on clinical and radiological evaluations. Specifically, for this purpose, follow-up contrast-enhanced T1w and T2-TSE sequences were reviewed. Response Assessment in Neuro-Oncology (RANO) [43] criteria were also evaluated to discriminate BRN from tumor progression in uncertain cases.

Based on CCC thresholds, radiomics features extracted from the reference tissue (gwm) under the optimal configuration were stratified into three subsets: baseline (all features), ≥good (CCC ≥ 0.70), and excellent (CCC ≥ 0.85) features. The 0.70 threshold provides a baseline of acceptable reproducibility without excessive information loss, while a stricter 0.85 cutoff maximizes generalizability but risks discarding predictive features. Within each subset, features were subjected to unsupervised filtering, including near-zero variance removal (threshold = 10^−6^) and redundancy reduction (i.e., features with mean Spearman correlation ≥ 0.85 were discarded).

The Wilcoxon rank-sum test was used for feature selection. Adhering to the one-feature-per-10-events rule [44], the top-4 and top-2 features were selected from the heterogeneous and homogeneous groups, respectively. Finally, a multivariable logistic regression (LR) classifier was used for outcome prediction. The predictive performance was quantified using the micro-averaged area under the Receiver Operating Characteristic (ROC) curve (AUC). Following the recommendations of Tsamardinos et al. [45], this was measured within a 5-times-repeated stratified 5-fold cross-validation (CV) framework. Feature selection was strictly confined to the training folds to prevent data leakage, while predictive performance was measured exclusively on the test fold to avoid optimistic bias; the final signature, however, was developed using the entire dataset. As noted by Tsamardinos et al. [45], for small datasets, this repeated multi-fold CV protocol without leakage yields an unbiased estimate, which can serve as a proxy for the expected performance of the final signature derived from the entire dataset.

The statistical significance of the observed AUC was assessed using the Wilcoxon rank-sum test [46], while bootstrap resampling (n = 5000) was used to estimate the 95% CI. ROC curves were compared using DeLong’s test, with Benjamini–Hochberg correction for multiple comparisons. The level of statistical significance was set at α = 0.05. Finally, to compare the selected signatures, maximum weighted matching based on the Spearman correlation coefficient was performed [47,48].

## 3. Results

SynthStrip, FreeSurfer’s MR sequence-agnostic method, demonstrated the best skull-stripping performance, while FSL’s FAST proved to be the most effective for tissue segmentation.

### 3.1. Stability Analysis

A total of 52 paired follow-up FS and non-FS radiological scans were available for the stability analysis (see Table S2). Among the 40 evaluated configurations, <brain BFC, no Normalization; Combat> demonstrated the highest median CCC of 0.58 (95% CI = 0.51–0.66) for the reference gwm features. This was closely followed by the <no BFC, no Normalization; Combat> configuration with a median CCC of 0.57 (0.50–0.65). Pairwise comparison revealed no statistically significant difference between these two processing pipelines (*p* = 1.0). Statistical similarity to the top performer was also observed across configurations combining brain normalization with most BFC variants (none, brain, or gwm; *p* > 0.05), except for the default BFC (*p* < 0.001). Similarly, the configuration <gwm BFC, no Normalization; Combat> also demonstrated statistical equivalence (*p* = 1.0). Tables S3 and S4 in the Supplementary Materials summarize these results. Based on the principle of parsimony, the simplest statistically equivalent processing pipeline, <no BFC, no Normalization; Combat>, was selected as the optimal processing configuration.

**Figure 2:**
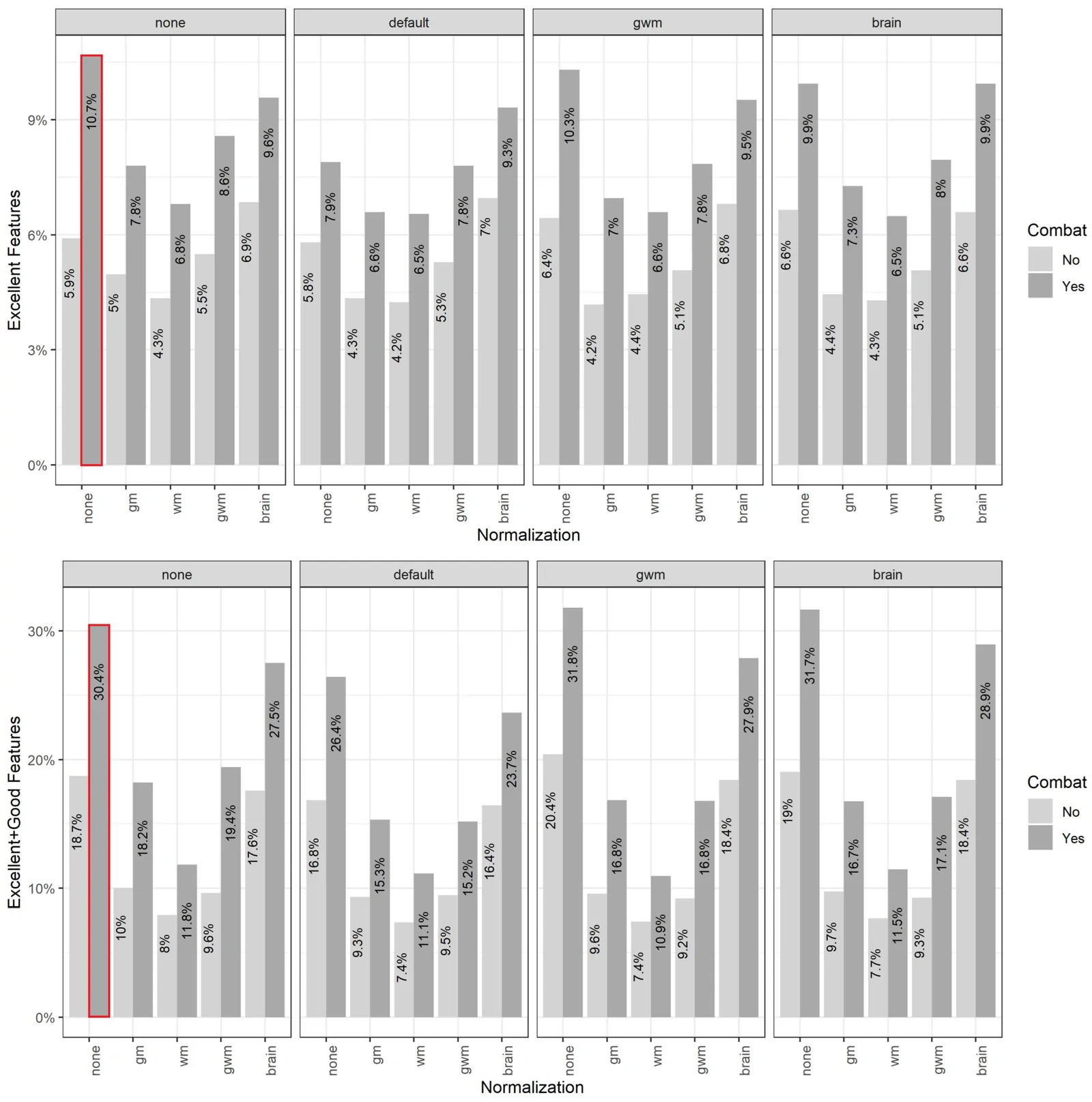
Percentages of gwm radiomics features for stability levels—at least good (CCC ≥ 0.7) and excellent (CCC ≥ 0.85)—for each image-level processing and harmonization choice. The selected optimal configuration appears as bolded red bars.

These findings were strongly supported by the multivariable linear mixed-effects regression analysis (see Supplementary Materials, Table S5). The regression demonstrated that Combat harmonization positively influenced gwm feature stability (β = 0.12, *p* < 0.001), whereas, on average, image-level processing methods impacted negatively (β = −0.16 to −0.003, *p* < 0.05). Consequently, the reference level (i.e., no Normalization, no BFC) with Combat emerged as the optimal configuration. As previously observed, the regression also highlighted similar top-performers. Within normalization variants, Z-score transformation relying on brain tissue (Z_brain_: β = −0.0096, *p* < 0.001) was highly competitive regardless of the chosen BFC. Conversely, in the absence of normalization, BFC utilizing gwm (β = −0.007, *p* < 0.001) was a strong contender. Figure 2 presents the percentage of gwm features for stability levels—at least good (≥good) and excellent—for each image-level processing and harmonization choice.

**Figure 3:**
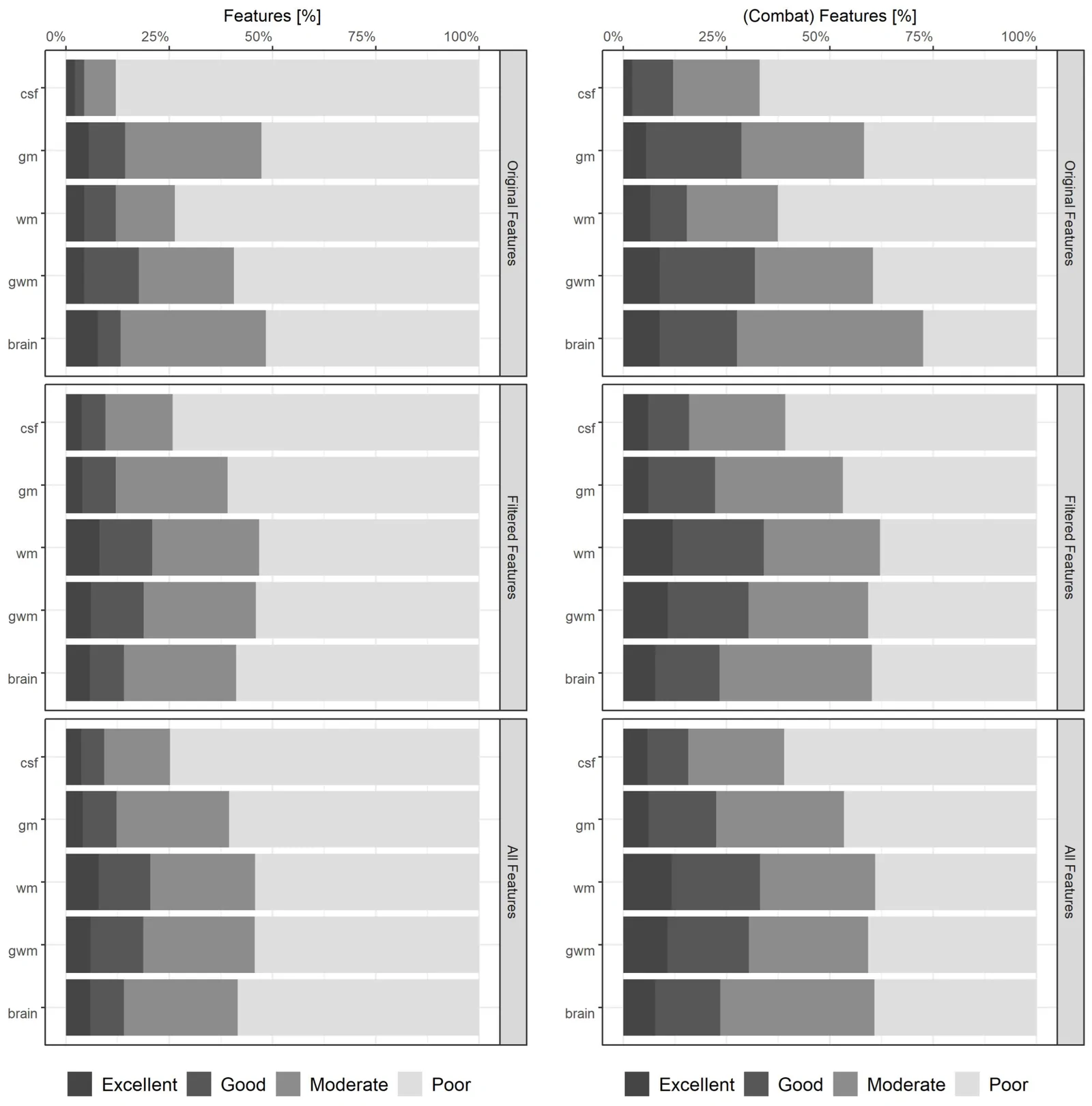
Stability profile of features extracted from each segmented tissue, linked to the optimal image-level processing method, stratified based on the application of Combat.

Focusing exclusively on the optimal configuration, the fraction of gwm features exhibiting excellent-level stability was relatively high (% CCC_excellent_ = 10.7). Although Combat consistently increased stability, the relative improvement in the excellent feature fraction was lowest for the optimal image-level processing choice (+55%), whereas it ranged from +60–70% for other configurations. In this setup, the remaining CCC levels—good, moderate, and poor—accounted for 19.7%, 28.9%, and 40.7% of gwm features, respectively, with nearly 30% of features showing ≥good stability (see Table 2). The gwm features that demonstrated at least good- and excellent-level stability for the optimal configuration are listed in Supplementary Materials, Table S6. Figure 3 illustrates the stability profile of features extracted from each segmented tissue, linked to the optimal image-level processing method, stratified based on the application of Combat.

Regression analysis using the key acquisition parameter difference in FS and non-FS sequences, TR (ΔTR range: [−9840, 4030]), showed no statistically significant effect on the stability of gwm features. Detailed results are provided in Supplementary Materials, Section S1.

**Figure 4:**
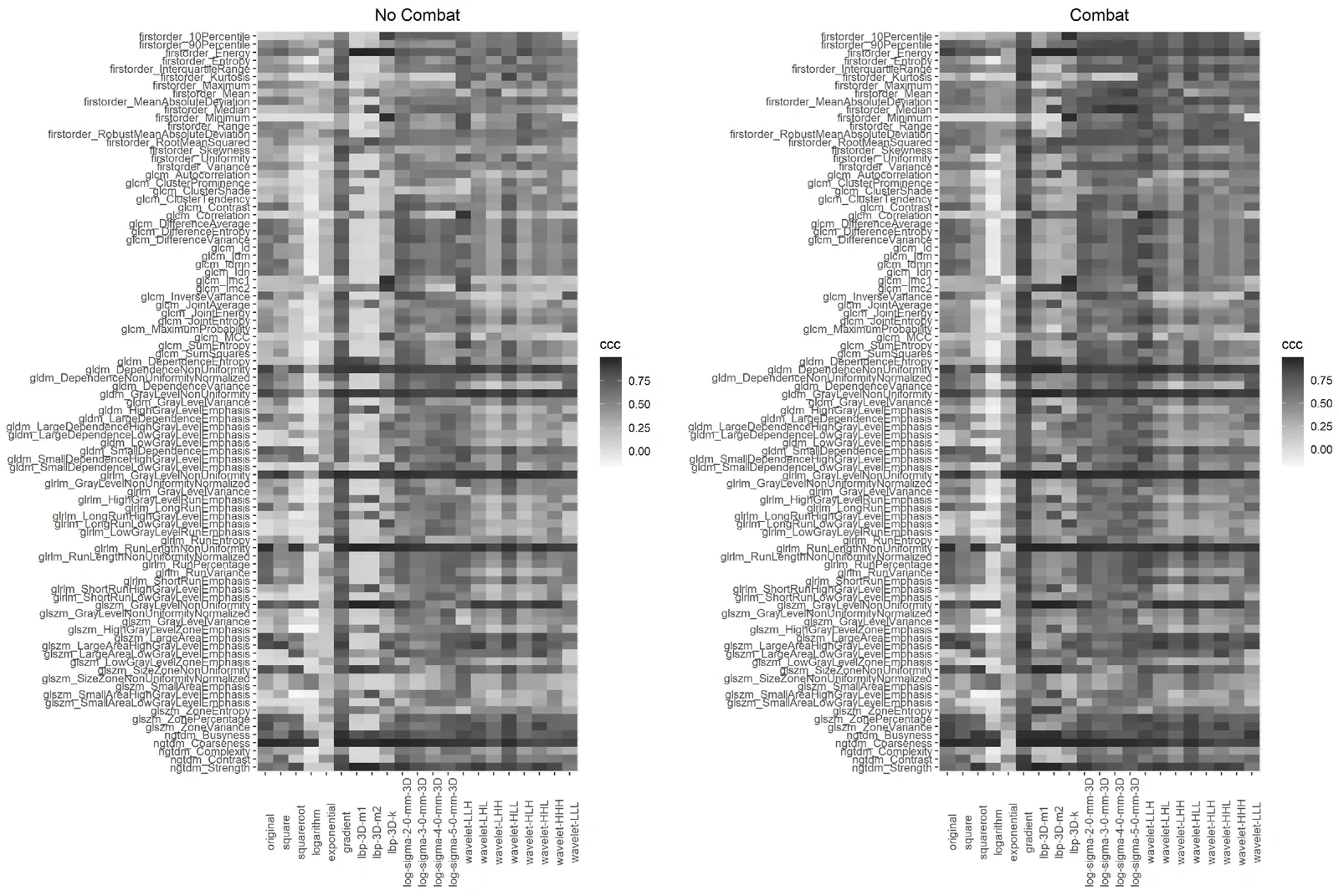
Concordance correlation coefficient of gray and white matter features associated with the optimal image-level processing strategy, grouped by image filtering choice and feature family, and further stratified based on the utilization of Combat.

Figure 4 reports the CCC of gwm features associated with the best image-level processing strategy, grouped by the image filter choice, and feature family, stratified further based on the utilization of Combat. Details on the brain tissues and filter-specific stability are reported in the Supplementary Materials, Section S2.

### 3.2. Radiomics Modeling

A description of both the heterogeneous and homogeneous cohorts is provided in Supplementary Tables S7–S9. Within a median follow-up time of 23 (IQR: 19–37) and 23 (19–27) months, BRN was reported for 32 (prevalence = 40%; n_G1_ = 22; n_G2_ = 10) and 12 patients (34%; n_G1_ = 8; n_G2_ = 4), in the heterogeneous and homogeneous cohorts, respectively. Of note, a longer follow-up time characterized patients experiencing BRN compared to negative cases (*p* ≤ 0.002; see Tables S7 and S8). Significant differences across FS (see Table S9) were reported for histology (*p* = 0.002), while a trend toward significance was noted for BRN ≥ G1 (*p* = 0.068).

As detailed in Table 2, radiomics features extracted from the reference tissue (gwm) underwent substantial reduction based on CCC thresholds. Compared to the baseline (n = 1911), thresholding based on CCC yielded an average feature space reduction of approximately 70% (581) when considering ≥good features, and nearly 90% (204) when restricted to only excellent features. The predictive signatures derived from the heterogeneous and homogeneous cohorts, stratified based on CCC thresholds, are listed in Table S10.

Table 3 summarizes the predictive performance of the selected signatures across MR subgroups and stability thresholds. Although the AUC measures were low to moderate (AUC range: 0.49–0.70), applying stricter CCC thresholds generally resulted in an increase in the AUC values of the LR classifier across all data subgroups (ΔAUC range: 0.02–0.19). When restricting the feature pool to only include ≥good features, the performance changes relative to the baseline were modest or mixed (−0.02 to +0.08), whereas a steeper, consistent improvement was observed with excellent features (0.05–0.19). Specifically, in the heterogeneous cohort, compared to the baseline (AUC = 0.52, 95% CI = 0.50–0.54; *p* = 0.081), a steady but modest performance gain was observed both with ≥good features (0.54, 0.52–0.56; *p* < 0.001), and excellent features (0.57, 0.55–0.59; *p* < 0.001). Within the homogeneous cohorts, the performance gains were most pronounced in the non-FS cohort with excellent features (baseline: 0.51, 0.48–0.54; *p* = 0.593 vs. excellent: 0.70, 0.69–0.73; *p* < 0.001). Conversely, the FS group exhibited a slight performance drop with ≥good features (baseline: 0.52, 0.48–0.55; *p* = 0.455 vs. ≥good: 0.49, 0.46–0.52; *p* = 0.521) before improving to 0.57 (0.54–0.60; *p* < 0.001) with excellent features. Delong’s test revealed that the AUC difference between baseline and excellent subsets was statistically significant across both homogeneous and heterogeneous cohorts (*p* ≤ 0.004). On the other hand, the difference in predictive performance of signatures derived using excellent features in the homogeneous FS and non-FS cohorts was not statistically significant (*p* = 0.345).

Regarding correlations (see Figure S2, in Supplementary Materials), in the heterogeneous cohort, the baseline signature exhibited moderate correlation with the signatures derived from CCC-thresholded subsets (Spearman range: 0.64–0.66). Conversely, the signatures derived from ≥good and excellent pools shared a strong correlation (0.86). In the homogeneous cohorts, strong sequence-specific signature dependencies were observed. Specifically, signatures derived from within the same sequence subgroup demonstrated moderate to near perfect correlation (FS: 0.68; non-FS: 0.61–0.96), whereas, across homogeneous subgroups (FS vs. non-FS), correlations remained low to moderate (0.27–0.69). Finally, between cohorts, while the heterogeneous signature generally demonstrated moderate to high correlations with signatures derived from the homogeneous non-FS subgroup (0.47–1.0), the correlation was low to moderate with the FS subgroup (0.27–0.42). In both cases, correlations were relatively higher (0.42–1.0), while considering only restricted CCC-subsets (≥good or excellent).

## 4. Discussion

MRI sequences are often tailored towards specific lesions and are subjected to variations. Whether visible or not, it is important to quantify the impact of such differences on radiomics feature stability and in downstream radiomics applications. In this study, we evaluated the influence of chemical shift-based fat saturation on the stability of T2-TSE radiomics features extracted from skull-base brain tissues. This is clinically relevant, as T2-TSE scans are often acquired with a mixed fat saturation protocol, yet they could be useful for developing multimodal, radiomics-based BRN prediction models in skull-base proton therapy. However, the direct integration of such mixed imaging features may confound signature development. To overcome this, we identified features stable to this protocol variation and explored their contribution to supporting the development of a BRN radiomics model. To provide a technical framework, we also investigated popular image- and feature-level processing schemes and assessed their impact on stability. Stable features corresponding to the optimal processing configuration were subsequently applied to patients with FS and/or non-FS T2-TSE scans to predict proton therapy-induced BRN, ultimately exploring whether prioritizing stability preserves clinically relevant information.

Overall, low feature stability (≥good: 30%, excellent: 10.7%) was recorded even with the optimal empirically selected T2w MRI processing configuration. Although literature suggests high reproducibility for T2w (91%/56%, intra-/inter-scanner) compared to other sequences, such as T1w (92%/2%) and diffusion weighted imaging (38%/15%) in intra-/inter-scanner settings [49], significantly low stability was reported when protocols variations (e.g., TR/TE) were present [11]. In our population, although TR was, on average, higher in the non-FS sequence, regression analysis suggested that it did not impact feature stability. This may be due to the consistent TE parameter present in our dataset, which is reported to have a greater impact on feature reproducibility than TR [11]. The inherent variations, derived from the difference in the fat signal present in the input sequences, may be a relevant contributor to such low stability. For instance, the myelin lining nerve fibers in the wm is characterized by a high percentage of lipids, hence fat suppression may increase the relative signal intensities, which also explains the markedly low stability observed among first-order features in our analysis.

Several processing configurations emerged as equally effective at improving the stability of the reference tissue (gwm) features, including Combat harmonization applied without image-level processing. Since no single configuration demonstrated a significant performance advantage (*p* > 0.05), the simplest configuration (<no BFC, no Normalization; Combat>) was selected as the optimal choice based on the principle of parsimony. This choice may be counterintuitive, especially since radiomics studies often highlight the importance of image-level processing techniques in MR images to improve stability [50]. However, the majority of these studies examine feature stability across independent acquisitions, where properties such as the device manufacturer or hardware setup differ and are expected to be the main source of instability. In contrast, our study investigated the T2-TSE scans that differed in fat saturation, acquired during the same MR examination, thus eliminating the hardware variability alongside potential acquisition-related confounders that primarily affect both MR field heterogeneity and the signal intensity distribution. In addition, texture features constituted the largest fraction of stable excellent features. Carré et al. [14], while assessing the impact of image-level processing techniques on brain T1w with Gadolinium contrast-enhancement and T2w-FLAIR, observed that for radiomics studies involving texture features alone, image-level processing strategies may have little to no impact. Instead, feature-level processing methods like Combat harmonization improves stability regardless of image-level processing choices [18,51]. Li et al. [52], while assessing the impact of the image- and feature-level processing techniques on T1w images, observed that Combat is vital in removing variations unrelated to biology. In fact, they reported that methods like BFC, image resampling, and intensity normalization, when applied without Combat, had little to no effect in improving feature stability. Consistently, in our study, Combat played a key role, resulting in at least 55% improvement in the stability of excellent gwm features regardless of the choice of the image-level processing method.

In radiomics literature, the use of filtered features is known to enhance the overall performance of radiomics analysis [53,54]. However, IBSI (v1) does not address the reproducibility of radiomics features extracted from filter-derived images. In our analysis, wavelet- and gradient-filtered features demonstrated the highest stability. Since the majority of the features demonstrating excellent stability were textures, these image filters seem to affect mostly this feature family. On the other hand, considering first-order features alone, the LoG filter was more influential than wavelet. While several studies reported improvements in stability due to wavelet and LoG-filtered features [55,56], the gradient filter, being a new addition to the PyRadiomics filter repertoire, lacks sufficient literature support. Although not widely recognized, this boost in stability may be attributed to the denoising properties of these image filters. For instance, a convolutional image filter like LoG effectively substitutes raw voxel values with a weighted average of its local neighborhood, consequently reducing the influence of outliers. While more advanced denoising approaches exist, we limited our focus to image filters as they are most widely used in radiomics literature.

For the downstream radiomics modeling study, we gathered T2-TSE data with a mixed-FS protocol (n = 80, prevalence = 40%) to explore the impact of stable feature subsets selected from these heterogeneous sequences for the development of a brain radionecrosis prediction model. Given the explorative nature of this study, we limited our analysis to a binary classification task, solely using radiomics features. While integrating additional clinical and/or dosimetric parameters may be pivotal in developing a radiomics signature for BRN, doing so in this study would confound our efforts to disentangle the specific influence of FS. Additionally, using a very small, homogeneous subset derived from the same patients (n = 35, prevalence = 0.34), we investigated the differences in predictive performance and correlation patterns of radiomics signatures developed on homogeneous samples with those developed on heterogeneous ones.

While predictive signatures derived from the entire radiomics feature set (baseline, n = 1911) showed poor discriminative performance across both homogeneous and heterogeneous cohorts (AUC range: 0.51–0.52, *p* > 0.05), restricting the feature pool to highly stable “excellent” features (~90% reduction in features) consistently improved AUC (0.57–70, *p* < 0.001). This suggests that the CCC-based feature filtering is predominantly eliminating noisy features and captures biological signal rather than protocol-specific characteristics, thereby improving the signal-to-noise ratio necessary for model development. The greatest performance gain was observed in the homogeneous non-FS subgroup (ΔAUC = 0.19), compared to moderate improvements in both the FS-only and mixed cohorts (ΔAUC = 0.05), suggesting that feature instability is sequence-dependent. Biologically, this may be due to the loss of important signal caused by fat tissue suppression in FS images. Conversely, the standard non-FS sequence captures a broader spectrum of biological information, which likely provides a richer feature space for classification. In fact, the correlation analyses revealed that signatures derived from the heterogeneous cohort using excellent features were perfectly concordant with the non-FS subgroup (Spearman: 1.0), whereas their correlation with the FS subgroup was moderate (0.42), hinting that the predictive signal is predominantly driven by stable non-FS features. It is also important to point out that this performance gap may also stem from errors in automated tissue segmentation. Given that the FSL FAST segmentation algorithm is optimized for standard (non-FS) T2-weighted images, inaccuracies in segmenting FS images may have confounded the radiomic analysis. Nevertheless, DeLong’s test revealed no statistically significant difference in AUC between these signatures (FS vs. non-FS; *p* = 0.345). Of note, this lack of significance may be attributed to our small sample size (n = 35), which limits statistical power.

Although the overall predictive performance of the best models remained low-to-moderate (AUC range: 0.57–0.70, *p* < 0.001), their statistical significance demonstrates genuine discrimination. However, their effect size is too small to reliably classify BRN on their own. Radiomics features should therefore be integrated into a multimodal radiomics-based NTCP model with additional clinical and treatment-related variables, after careful collinearity evaluation. Specifically, recent literature suggests that relative biological effectiveness-weighted dose and dose-averaged linear energy transfer-derived variables are key predictors in brain radionecrosis prediction [57,58].

Finally, we acknowledge the main limitations of our study and highlight future research directions. First, the retrospective, single-center design and small sample size constrained both a well-controlled feature reproducibility analysis and downstream radiomics modeling. Specifically, while the paired design of the stability study aimed to isolate the effect of FS, even the slightest protocol variations within our cohort may have influenced feature stability besides FS. Additionally, in the modeling cohorts, the underlying clinical characteristics remained imbalanced. Furthermore, the rarity of high-grade BRN restricted our endpoint to any occurrence (G1+) rather than clinically relevant symptomatic (G2+) BRN. Multicenter studies with adequate sample size, statistical analysis and proper experimental settings should be envisioned to investigate the impact of fat saturation coupled with common protocol variations for verifying the actual implications of stability findings in downstream modeling. Second, although developing a BRN signature was beyond the scope of this study, our conclusions are further limited by several methodological considerations. Applying Combat harmonization to a cohort with patient overlap risked data leakage. However, utilizing different scan time points (follow-up vs. baseline) and observing comparable performance in the unharmonized homogeneous cohorts suggest minimal bias. Moreover, our study may be confounded by automated FSL segmentation errors (especially in FS scans), the inclusion of pathological tissue in modeling, and the absence of a time-to-event analysis. Third, our image-level processing methods ignored tissue-level heterogeneity. Lastly, our downstream radiomics modeling analysis focused exclusively on BRN prediction following skull-base proton therapy and may not generalize to other clinical outcomes.

Indeed, due to the limited stability over FS in T2-TSE, we strongly believe that solutions to improve reproducibility should also be explored. Generative deep learning models, having shown tremendous success in improving and generating good-quality synthetic images, could offer a viable alternative for synthesizing MR data [59,60] in this context and are worth investigating as a future direction [61,62]. These models may be leveraged to transform heterogeneous images into a single modality of choice, facilitating the creation of comprehensive homogeneous datasets preserving inherent tissue signal.

## 5. Conclusions

Our findings urge caution when merging T2-TSE images that differ in fat saturation to enlarge the dataset for radiomics applications involving skull-base brain tissue. In the context of BRN prediction using heterogeneous FS data, enforcing stability-based thresholds is an essential methodological step that not only offsets protocol-induced variability but also actively improves predictive performance. Finally, given the overall low stability of radiomics features, efforts should be made to establish common protocols and clearer imaging guidelines. While this study offers a promising framework, our findings are preliminary and require rigorous validation in future studies before definitive conclusions can be drawn.

## Figures and Tables

**Table 1:** Description of the main acquisition T2-TSE parameters for the fat-saturated and non-fat-saturated protocols.

	T2-Weighted Turbo Spin Echo	
	Fat-Saturated ^1^	Non Fat-Saturated ^1^
TR [ms]	5580.00 (5300.00, 6342.50)	6375.00 (6010.00, 8470.00)
TE [ms]	104.00 (104.00, 104.00)	104.00 (104.00, 104.00)
Voxel Spacing-x [mm]	0.47 (0.47, 0.47)	0.47 (0.47, 0.47)
Voxel Spacing-y [mm]	0.47 (0.47, 0.47)	0.47 (0.47, 0.47)
Voxel Spacing-z [mm]	3.30 (3.30, 3.30)	3.30 (3.30, 3.30)
Slice Thickness [mm]	3.00 (3.00, 3.00)	3.00 (3.00, 3.00)
Image Matrix-x	512.00 (512.00, 512.00)	512.00 (512.00, 512.00)
Image Matrix-y	512.00 (512.00, 512.00)	512.00 (512.00, 512.00)
Echo Train length	20.00 (20.00, 20.00)	20.00 (20.00, 20.00)
Number Of Averages	3.00 (3.00, 3.00)	1.00 (1.00, 2.00)
Flip Angle [°]	80.00 (80.00, 80.00)	80.00 (80.00, 80.00)

^1^ Median (IQR).

**Table 2:** Description of CCC-based excellent, good, moderate, and poor radiomics feature fractions for gwm, with the optimal image-level processing configuration.

		No Combat [% (N)]				Combat [% (N)]			
		Excellent	Good	Moderate	Poor	Excellent	Good	Moderate	Poor
**Original** (n = 91)	*Overall*	4.4% (4)	13.2% (12)	23.1% (21)	59.3% (54)	8.8% (8)	23.1% (21)	28.6% (26)	39.6% (36)
	*First Order*	0.0% (0)	0.0% (0)	4.4% (4)	14.3% (13)	1.1% (1)	1.1% (1)	12.1% (11)	4.4% (4)
	*Texture*	4.4% (4)	13.2% (12)	18.7% (17)	45.1% (41)	7.7% (7)	22.0% (20)	16.5% (15)	35.2% (32)
**Filtered** (n = 1820)	*Overall*	6.0% (109)	12.8% (233)	27.1% (494)	54.1% (984)	10.8% (196)	19.6% (356)	28.9% (526)	40.8% (742)
	*First Order*	0.3% (5)	2.1% (38)	6.4% (116)	9.9% (181)	1.2% (22)	6.1% (111)	5.8% (106)	5.5% (101)
	*Texture*	5.7% (104)	10.7% (195)	20.8% (378)	44.1% (803)	9.6% (174)	13.5% (245)	23.1% (420)	35.2% (641)
**Original+Filtered** (n = 1911)	*Overall*	5.9% (113)	12.8% (245)	26.9% (515)	54.3% (1038)	10.7% (204)	19.7% (377)	28.9% (552)	40.7% (778)
	*First Order*	0.3% (5)	2.0% (38)	6.3% (120)	10.2% (194)	1.2% (23)	5.9% (112)	6.1% (117)	5.5% (105)
	*Texture*	5.7% (108)	10.8% (207)	20.7% (395)	44.2% (844)	9.5% (181)	13.9% (265)	22.8% (435)	35.2% (673)

**Table 3:** Discriminative Performance of the LR BRN classifier in the heterogeneous and homogeneous cohorts, for baseline (all features), at least good (CCC ≥ 0.70), and only excellent (CCC ≥ 0.85) features.

CCC Threshold	Heterogeneous Cohort ^1^ [Top-4 Features] (Prevalence = 40%)	Homogeneous FS Cohort ^1^ [Top-2 Features] (Prevalence = 34%)	Homogeneous Non-FS Cohort ^1^ [Top-2 Features] (Prevalence = 34%)
**baseline** (n = 1911)	0.52 [0.50, 0.54] (0.081)	0.52 [0.48, 0.55] (0.455)	0.51 [0.48, 0.54] (0.593)
**≥good** (n = 581)	0.54 [0.52, 0.56] (<0.001)	0.49 [0.46, 0.52] (0.521)	0.59 [0.56, 0.62] (<0.001)
**excellent** (n = 204)	0.57 [0.55, 0.59] (<0.001)	0.57 [0.54, 0.60] (<0.001)	0.70 [0.69, 0.73] (<0.001)

^1^ LR AUC [95% CI] (*p*-value).

## Data Availability

The raw data supporting the conclusions of this article will be made available by the authors upon reasonable request.

## Supplementary Materials

The following supporting information can be downloaded at: https://www.mdpi.com/article/10.3390/cancers18162636/s1, Table S1: List of the 40 evaluated image- and feature-level configurations; Table S2: Description of the study sample for stability analysis; Table S3: Median CCC and 95% confidence interval associated with 40 processing configurations investigated in this study; Table S4: Pairwise comparison of each configuration with <brain BFC, no Normalization; Combat> using the Mann–Whitney U-test; Table S5: Impact of image- and feature-level processing configurations on the stability of gwm features are summarized in the tables: (i) all v/s filtered v/s original, and (ii) all v/s first-order v/s textureb; Table S6: List of the gray and white matter features that demonstrated at least good and excellent-level stability for the best processing configuration; Table S7: Description of the heterogeneous sample to develop a model for brain radionecrosis prediction after skull-base proton therapy, grouped by skull-base brain necrosis ≥ G1; Table S8: Description of the homogeneous sample to develop a model for brain radionecrosis prediction after skull-base proton therapy, grouped by skull-base brain necrosis ≥ G1; Table S9: Description of the heterogeneous sample to develop a model for brain radionecrosis prediction after skull-base proton therapy, grouped by FS and non-FS in T2-TSE imaging sequences; Table S10: List of radiomic features selected during BRN predictive modeling in the heterogeneous (mixed FS protocol) and homogeneous cohorts; Figure S1: Patient flow diagram describing the selection process of the two studies: stability study, and the pilot downstream radiomics modeling; Figure S2: Spearman correlation of radiomics signatures selected from the homogeneous FS, homogeneous non-FS, and heterogeneous (FS + non-FS) datasets at different CCC stability thresholds (baseline, ≥good, and excellent); Section S1: Impact of ΔTR on the reproducibility of gwm features for the best-processing configuration (no BFC, no normalization, with Combat); Section S2: Details on the brain tissue- and filter-specific stability.

## Abbreviations

The following abbreviations are used in this manuscript:

RT	Radiotherapy
NTCP	Normal Tissue Complication Probability
TCP	Tumor Control Probability
BRN	Brain Radionecrosis
MRI	Magnetic Resonance Imaging
T1w	T1-weighted
T2w	T2-weighted
FS	Fat saturation
BFC	Bias field correction
T2-TSE	T2-weighted turbo spin echo
3T	3 Tesla
TR	Time of Repetition
TE	Time of Echo
IQR	Inter-quartile range
FSL	FMRIB Software Library
FAST	FMRIB’s automated segmentation tool
BET	Brain extraction tool
ANTs	Advanced normalization tools
gm	Gray matter
wm	White matter
csf	Cerebro-spinal fluid
gwm	Gray and white matter
IBSI	Image biomarker standardization initiative
CCC	Concordance correlation coefficient
CTCAEv5	Common terminology for adverse events, version 5
RF	Random Forest
AUC	Area under the receiver operating characteristic curve
LOOCV	Leave-one-out cross-validation
LoG	Laplacian of gaussian
