# Supplementary material for: Exploring the Impact of T2-Weighted MRI Fat Saturation on Radiomics Stability for Brain Radionecrosis Prediction After Skull-Base Proton Therapy: A Pilot Study
Source: Cancers (Basel). 2026 Aug 15;18(16):2636. doi: 10.3390/cancers18162636 (PMC13511151; doi:10.3390/cancers18162636)
Supplement: Supplementary file 1 [file cancers-18-02636-s001.zip › Supplementary_Materials.pdf]

Exploring the Impact of T2-Weighted MRI Fat Saturation on Radiomics Stability for Brain Radionecrosis Prediction After Skull-Base Proton Therapy: A Pilot Study

Table S1. List of the 40 evaluated image- and feature-level configurations.

Configurations	**BFC**				**Normalization**					**Combat**	
	None	*Default*	*gwm*	*brain*	*None*	*gm*	*wm*	*gwm*	*brain*	*No*	*Yes*
1	x				x					x	
2	x				x						x
3	x					x				x	
4	x					x					x
5	x						x			x	
6	x						x				x
7	x							x		x	
8	x							x			x
9	x								x	x	
10	x								x		x
11		x			x					x	
12		x			x						x
13		x				x				x	
14		x				x					x
15		x					x			x	
16		x					x				x
17		x						x		x	
18		x						x			x
19		x							x	x	
20		x							x		x
21			x		x					x	
22			x		x						x
23			x			x				x	
24			x			x					x
25			x				x			x	
26			x				x				x
27			x					x		x	
28			x					x			x
29			x						x	x	
30			x						x		x
31				x	x					x	
32				x	x						x
33				x		x				x	
34				x		x					x
35				x			x			x	
36				x			x				x
37				x				x		x	
38				x				x			x
39				x					x	x	
40				x					x		x

Table S2. Description of the study sample for stability analysis.

	**N = 52**[1]
**Age [years]**	57.7 (48.6, 68.4)
**Gender**	
Female	24 (46%)
Male	28 (54%)
**Histology**	
Chordoma	52 (100%)
**Surgery**	
Yes	46 (88%)
**Total Number Of Surgeries**	1.0 (1.0, 2.0)
**Surgical Technique**	
Craniotomy	6 (11.5%)
Endoscopic endonasal approach	40 (77%)
**Hypertension**	11 (21%)
**Diabetes**	2 (0.04%)
**Baseline_Macroscopic_Disease**	34 (65.4%)
**GTV [cc]**	3.5 (0.0, 12.5)
**PT_Intent**	
Adjuvant	45 (86.5%)
Exclusive PT	1 (0.02%)

[1]Median (IQR); n (%)

Table S3. Median CCC and 95% confidence interval associated with 40 processing configurations investigated in this study.
See file: config_stats.csv

Table S4. Pairwise comparison of each configuration with <brain BFC, no Normalization; Combat> using the Mann–Whitney U-test.
See file: config_pvalues.csv

Table S5: Impact of image- and feature-level processing configurations on the stability of gwm features are summarized in the tables: (i) all v/s filtered v/s original, and (ii) all v/s first-order v/s textureb; BFC_x—Bias Field Correction (using x = default/gwm/brain), $Norm_x$—Normalization (using x = brain/gm/gwm/wm); statistically significant rows are in bold.
With respect to the reference tissue (gwm) radiomics features, a multivariable linear mixed-effect (random intercept for the feature and image filters) regression analysis was conducted to explore the influence of processing configurations (BFC, normalization, and Combat) on CCC, the metric of feature stability.

(i) all v/s filtered v/s original

Parameter	Subset	Estimate	95% Confidence Interval	P-value	Adj. P-value (FDR)
(Intercept)	[All features]	0.4455	[0.4363, 0.4546]	**<0.001**	-
	[Filtered]	0.4469	[0.4375, 0.4562]	**<0.001**	-
	[Original]	0.4176	[0.3735, 0.4616]	**<0.001**	-
BFC: brain	[All features]	-0.0026	[-0.0055, 3e-04]	0.0783	0.0783
	[Filtered]	-0.0029	[-0.0058, 1e-04]	0.0566	0.0566
	[Original]	0.0033	[-0.0083, 0.015]	0.5753	0.5753
BFC: default	[All features]	-0.0190	[-0.0219, -0.0161]	**<0.001**	**<0.001**
	[Filtered]	-0.0182	[-0.0212, -0.0153]	**<0.001**	**<0.001**
	[Original]	-0.0349	[-0.0466, -0.0233]	**<0.001**	**<0.001**
BFC: gwm	[All features]	-0.0072	[-0.0101, -0.0044]	**<0.001**	**<0.001**
	[Filtered]	-0.0069	[-0.0098, -0.0039]	**<0.001**	**<0.001**
	[Original]	-0.0148	[-0.0264, -0.0031]	0.0134	0.0151
Normalization: brain	[All features]	-0.0096	[-0.0129, -0.0064]	**<0.001**	**<0.001**
	[Filtered]	-0.0065	[-0.0098, -0.0032]	**<0.001**	**<0.001**
	[Original]	-0.0730	[-0.0861, -0.06]	**<0.001**	**<0.001**
Normalization: gm	[All features]	-0.1020	[-0.1052, -0.0988]	**<0.001**	**<0.001**
	[Filtered]	-0.0997	[-0.103, -0.0964]	**<0.001**	**<0.001**
	[Original]	-0.1482	[-0.1613, -0.1351]	**<0.001**	**<0.001**
Normalization: gwm	[All features]	-0.0809	[-0.0841, -0.0776]	**<0.001**	**<0.001**
	[Filtered]	-0.0793	[-0.0826, -0.0759]	**<0.001**	**<0.001**
	[Original]	-0.1129	[-0.126, -0.0999]	**<0.001**	**<0.001**
Normalization: wm	[All features]	-0.1629	[-0.1661, -0.1597]	**<0.001**	**<0.001**
	[Filtered]	-0.1624	[-0.1657, -0.1591]	**<0.001**	**<0.001**
	[Original]	-0.1730	[-0.186, -0.1599]	**<0.001**	**<0.001**
Combat: Yes	[All features]	0.1213	[0.1192, 0.1233]	**<0.001**	**<0.001**
	[Filtered]	0.1201	[0.118, 0.1222]	**<0.001**	**<0.001**
	[Original]	0.1455	[0.1372, 0.1538]	**<0.001**	**<0.001**

(ii) all v/s first-order v/s texture

Parameter	Subset	Estimate	95% Confidence Interval	P-value	Adj. P-value (FDR)
(Intercept)	[All features]	0.4455	[0.4363, 0.4546]	**<0.001**	-
	[First-Order]	0.4430	[0.4268, 0.4593]	**<0.001**	-
	[Texture]	0.4460	[0.4355, 0.4566]	**<0.001**	-
BFC: brain	[All features]	-0.0026	[-0.0055, 3e-04]	0.0783	0.0783
	[First-Order]	0.0010	[-0.0059, 0.0078]	0.7836	0.7836
	[Texture]	-0.0034	[-0.0065, -3e-04]	0.034	0.034
BFC: default	[All features]	-0.0190	[-0.0219, -0.0161]	**<0.001**	**<0.001**
	[First-Order]	-0.0092	[-0.016, -0.0023]	0.0085	0.0109
	[Texture]	-0.0213	[-0.0244, -0.0181]	**<0.001**	**<0.001**
BFC: gwm	[All features]	-0.0072	[-0.0101, -0.0044]	**<0.001**	**<0.001**
	[First-Order]	-0.0053	[-0.0121, 0.0015]	0.1286	0.1447
	[Texture]	-0.0077	[-0.0108, -0.0046]	**<0.001**	**<0.001**
Normalization: brain	[All features]	-0.0096	[-0.0129, -0.0064]	**<0.001**	**<0.001**
	[First-Order]	-0.0935	[-0.1012, -0.0859]	**<0.001**	**<0.001**
	[Texture]	0.0096	[0.0061, 0.0131]	**<0.001**	**<0.001**
Normalization: gm	[All features]	-0.1020	[-0.1052, -0.0988]	**<0.001**	**<0.001**
	[First-Order]	-0.1888	[-0.1964, -0.1812]	**<0.001**	**<0.001**
	[Texture]	-0.0820	[-0.0856, -0.0785]	**<0.001**	**<0.001**
Normalization: gwm	[All features]	-0.0809	[-0.0841, -0.0776]	**<0.001**	**<0.001**
	[First-Order]	-0.1585	[-0.1661, -0.1509]	**<0.001**	**<0.001**
	[Texture]	-0.0630	[-0.0665, -0.0595]	**<0.001**	**<0.001**
Normalization: wm	[All features]	-0.1629	[-0.1661, -0.1597]	**<0.001**	**<0.001**
	[First-Order]	-0.2403	[-0.2479, -0.2327]	**<0.001**	**<0.001**
	[Texture]	-0.1451	[-0.1486, -0.1416]	**<0.001**	**<0.001**
Combat: Yes	[All features]	0.1213	[0.1192, 0.1233]	**<0.001**	**<0.001**
	[First-Order]	0.1570	[0.1522, 0.1618]	**<0.001**	**<0.001**
	[Texture]	0.1131	[0.1108, 0.1153]	**<0.001**	**<0.001**

Table S6. List of the gray and white matter features that demonstrated at least good and excellent-level stability for the best processing configuration.
See file: stable_features.xlsx

Table S7. Description of the heterogeneous sample to develop a model for brain radionecrosis prediction after skull-base proton therapy, grouped by skull-base brain necrosis ≥ G1.

		**Skull-Base Brain Necrosis≥G1**		
	**Overall**, N = 80[1]	**No**, N = 48[1]	**Yes**, N = 32[1]	**p-value**[2]
**Age [years]**	56.6 (44.9, 66.6)	54.3 (44.4, 66.3)	59.4 (46.7, 67.1)	0.581
**Gender**				0.648
Female	40 (50%)	23 (48%)	17 (53%)	
Male	40 (50%)	25 (52%)	15 (47%)	
**Histology**				0.242
Chondrosarcoma	15 (19%)	11 (23%)	4 (13%)	
Chordoma	65 (81%)	37 (77%)	28 (88%)	
**Surgery**	79 (99%)	47 (98%)	32 (100%)	>0.999
**Total Number Of Surgeries**	1.0 (1.0, 2.0)	1.0 (1.0, 2.0)	1.0 (1.0, 2.0)	0.437
**Surgical Technique**				0.161
Craniotomy	16 (20%)	12 (25%)	4 (13%)	
Endoscopic endonasal approach	62 (78%)	34 (71%)	28 (88%)	
Endoscopic endonasal approach + Craniotomy	2 (2.5%)	2 (4.2%)	0 (0%)	
**Hypertension**	19 (24%)	12 (26%)	7 (22%)	0.670
**Diabetes**	4 (5.1%)	1 (2.2%)	3 (9.4%)	0.300
**Baseline_Macroscopic_Disease**	61 (76%)	38 (79%)	23 (72%)	0.453
**GTV [cc]**	4.5 (0.4, 14.4)	4.1 (0.6, 16.0)	4.6 (0.4, 12.7)	0.521
**PT_Intent**				0.514
Adjuvant	78 (98%)	46 (96%)	32 (100%)	
Exclusive PT	2 (2.5%)	2 (4.2%)	0 (0%)	
**BRN CTCAE grade**				-
0	48 (60%)	48 (100%)	0 (0%)	
1	22 (28%)	0 (0%)	22 (69%)	
2	10 (13%)	0 (0%)	10 (31%)	
**Follow-up Time [months]**	35.7 (16.8, 76.8)	25.0 (11.7, 71.9)	60.0 (33.9, 81.1)	0.002
**sequence**				0.068
t2w_fs	40 (50%)	20 (42%)	20 (63%)	
t2w_nfs	40 (50%)	28 (58%)	12 (38%)	

[1]Median (IQR); n (%)

[2]Wilcoxon rank sum exact test; Pearson's Chi-squared test; Fisher's exact test; Wilcoxon rank sum test

Table S8. Description of the homogeneous sample to develop a model for brain radionecrosis prediction after skull-base proton therapy, grouped by skull-base brain necrosis ≥ G1.

		Skull-Base Brain Necrosis≥G1		
	**Overall**, N = 35[1]	**No**, N = 23[1]	**Yes**, N = 12[1]	**p-value**[2]
**Age [years]**	53.5 (41.5, 66.0)	49.0 (38.6, 66.0)	59.4 (42.3, 63.2)	0.595
**Gender**				0.288
Female	16 (46%)	12 (52%)	4 (33%)	
Male	19 (54%)	11 (48%)	8 (67%)	
**Histology**				0.434
Chondrosarcoma	10 (29%)	8 (35%)	2 (17%)	
Chordoma	25 (71%)	15 (65%)	10 (83%)	
**Surgery**	34 (97%)	22 (96%)	12 (100%)	>0.999
**Total Number Of Surgeries**	1.0 (1.0, 2.0)	1.0 (1.0, 2.0)	1.0 (1.0, 1.0)	0.101
**Surgical Technique**				0.237
Craniotomy	8 (23%)	7 (30%)	1 (8.3%)	
Endoscopic endonasal approach	25 (71%)	14 (61%)	11 (92%)	
Endoscopic endonasal approach + Craniotomy	2 (5.7%)	2 (8.7%)	0 (0%)	
**Hypertension**	9 (26%)	7 (32%)	2 (17%)	0.439
**Diabetes**	2 (5.9%)	0 (0%)	2 (17%)	0.118
**Baseline_Macroscopic_Disease**	28 (80%)	20 (87%)	8 (67%)	0.200
**GTV [cc]**	4.3 (0.5, 14.9)	6.4 (2.3, 27.0)	1.1 (0.0, 7.4)	0.133
**PT_Intent**				>0.999
Adjuvant	34 (97%)	22 (96%)	12 (100%)	
Exclusive PT	1 (2.9%)	1 (4.3%)	0 (0%)	
**BRN CTCAE grade**				-
0	23 (66%)	23 (100%)	0 (0%)	
1	8 (23%)	0 (0%)	8 (67%)	
2	4 (11%)	0 (0%)	4 (33%)	
**Follow-up Time [months]**	16.3 (11.2, 31.3)	11.8 (9.4, 20.8)	31.3 (27.0, 42.5)	<0.001

[1]Median (IQR); n (%)

[2]Wilcoxon rank sum exact test; Pearson's Chi-squared test; Fisher's exact test; Wilcoxon rank sum test

Table S9. Description of the heterogeneous sample to develop a model for brain radionecrosis prediction after skull-base proton therapy, grouped by FS and non-FS in T2-TSE imaging sequences.

		T2-TSE Sequence		
	**Overall**, N = 80[1]	**t2w_fs**, N = 40[1]	**t2w_nfs**, N = 40[1]	**p-value**[2]
**Age [years]**	56.6 (44.9, 66.6)	58.1 (50.5, 69.6)	53.2 (41.5, 65.3)	0.103
**Gender**				0.655
Female	40 (50%)	21 (53%)	19 (48%)	
Male	40 (50%)	19 (48%)	21 (53%)	
**Histology**				0.002
Chondrosarcoma	15 (19%)	2 (5.0%)	13 (33%)	
Chordoma	65 (81%)	38 (95%)	27 (68%)	
**Surgery**	79 (99%)	40 (100%)	39 (98%)	>0.999
**Total Number Of Surgeries**	1.0 (1.0, 2.0)	1.0 (1.0, 2.0)	1.0 (1.0, 2.0)	0.721
**Surgical Technique**				0.156
Craniotomy	16 (20%)	6 (15%)	10 (25%)	
Endoscopic endonasal approach	62 (78%)	34 (85%)	28 (70%)	
Endoscopic endonasal approach + Craniotomy	2 (2.5%)	0 (0%)	2 (5.0%)	
**Hypertension**	19 (24%)	10 (25%)	9 (24%)	0.892
**Diabetes**	4 (5.1%)	2 (5.0%)	2 (5.3%)	>0.999
**Baseline_Macroscopic_Disease**	61 (76%)	29 (73%)	32 (80%)	0.431
**GTV [cc]**	4.5 (0.4, 14.4)	4.5 (0.1, 13.1)	4.3 (0.8, 14.9)	0.697
**PT_Intent**				>0.999
Adjuvant	78 (98%)	39 (98%)	39 (98%)	
Exclusive PT	2 (2.5%)	1 (2.5%)	1 (2.5%)	
**Brain Radionecrosis ≥ G1**	32 (40%)	20 (50%)	12 (30%)	0.068
**BRN CTCAE grade**				0.185
**0**	48 (60%)	20 (50%)	28 (70%)	
**1**	22 (28%)	14 (35%)	8 (20%)	
**2**	10 (13%)	6 (15%)	4 (10%)	
**Follow-up Time [months]**	35.7 (16.8, 76.8)	74.7 (51.9, 88.0)	16.8 (10.7, 31.0)	<0.001

[1]Median (IQR); n (%)

[2]Wilcoxon rank sum exact test; Pearson's Chi-squared test; Fisher's exact test; Wilcoxon rank sum test

Table S10. List of radiomic features selected during BRN predictive modeling in the heterogeneous (mixed FS protocol) and homogeneous cohorts.

CCC Threshold	Heterogeneous Sample (signature=top-4 features)	Homogeneous Sample FS (signature=top-2 features)	Homogeneous Sample non-FS (signature=top-2 features)
**baseline** (# features = 1911)	wavelet-HHH_glcm_MCC, wavelet-HHH_ngtdm_Complexity, wavelet-HHL_gldm_DependenceEntropy, wavelet-LLH_gldm_GrayLevelNonUniformity	wavelet-HLH_glcm_InverseVariance, lbp-3D-k_firstorder_10Percentile	wavelet-LLL_glszm_LargeAreaHighGrayLevelEmphasis, lbp-3D-k_firstorder_10Percentile
**>=good** (# features = 581)	wavelet-LLH_gldm_GrayLevelNonUniformity, wavelet-LLH_gldm_DependenceEntropy, wavelet-LLH_firstorder_Kurtosis, squareroot_glszm_LargeAreaLowGrayLevelEmphasis	lbp-3D-k_firstorder_10Percentile, wavelet-LHL_ngtdm_Strength	original_glszm_LargeAreaHighGrayLevelEmphasis, lbp-3D-k_firstorder_10Percentile
**excellent** (# features = 204)	wavelet-LLH_gldm_GrayLevelNonUniformity, original_glszm_LargeAreaEmphasis, wavelet-LLH_glcm_SumEntropy, wavelet-HLL_firstorder_Kurtosis	lbp-3D-k_firstorder_10Percentile, wavelet-LHL_ngtdm_Strength	wavelet-LLH_gldm_GrayLevelNonUniformity, original_glszm_ZoneVariance

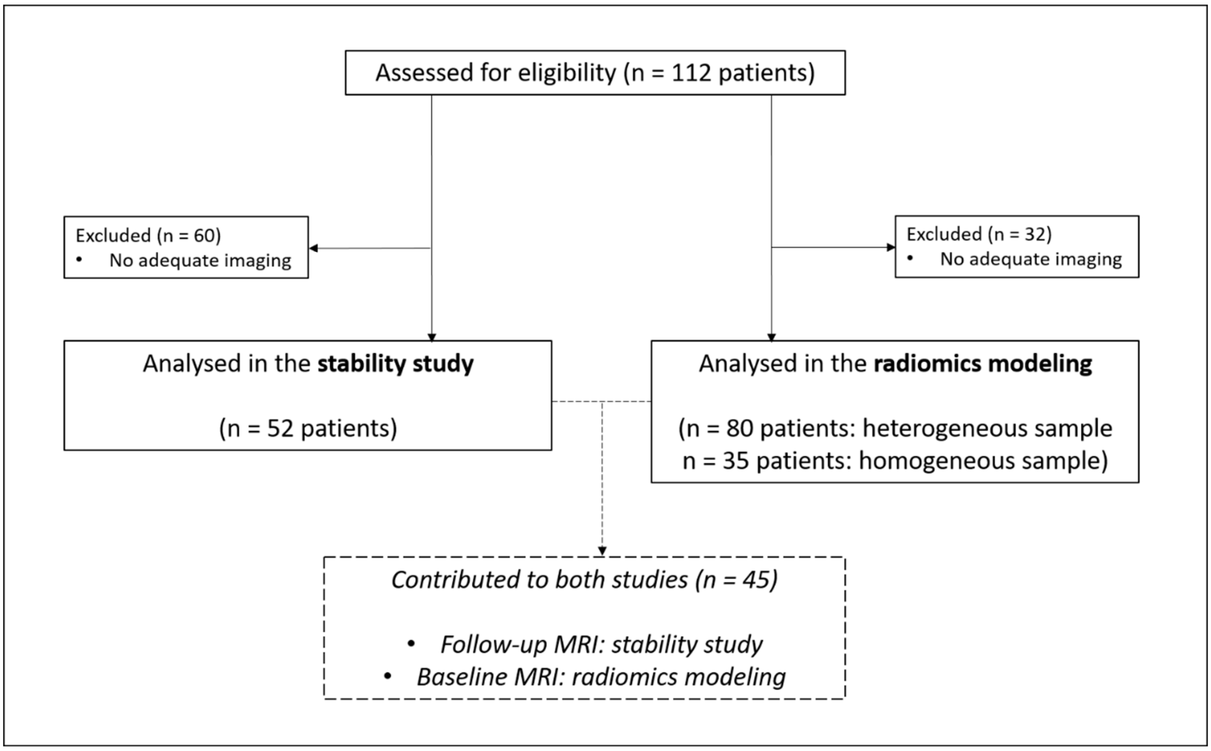

Figure S1. Patient flow diagram describing the selection process of the two studies: stability study, and the pilot downstream radiomics modeling.

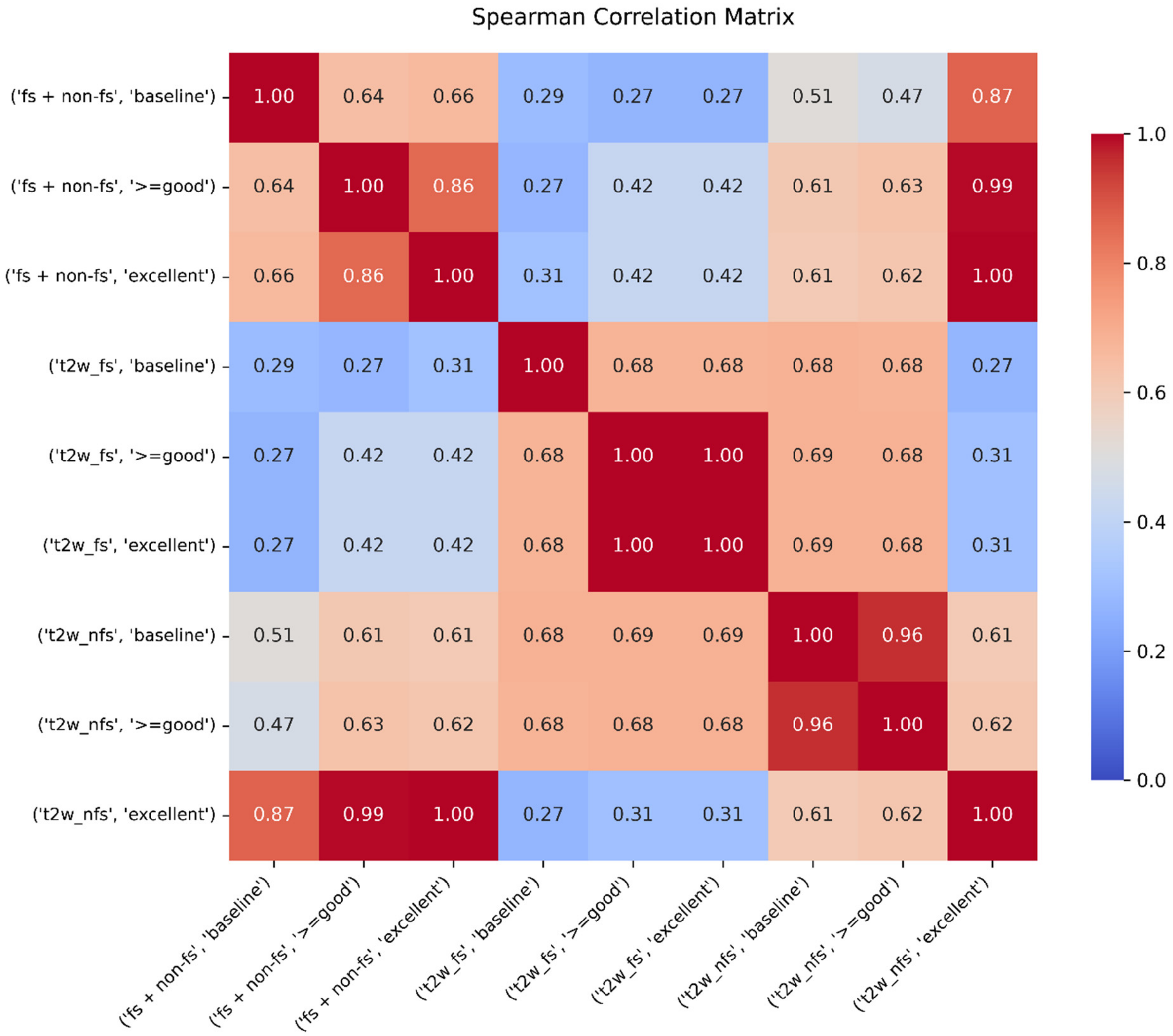

Figure S2. Spearman correlation of radiomics signatures selected from the homogeneous FS, homogeneous non-FS, and heterogeneous (FS + non-FS) datasets at different CCC stability thresholds (baseline, ≥good, and excellent)

Section S1: Impact of ΔTR on the reproducibility of gwm features for the best-processing configuration (no BFC, no normalization, with Combat)

$$\Delta TR = TR_{fs} - TR_{nfs}$$

The ΔTR values ranged from -9840.0 to 4030.0. The figure below displays the ΔTR histogram.

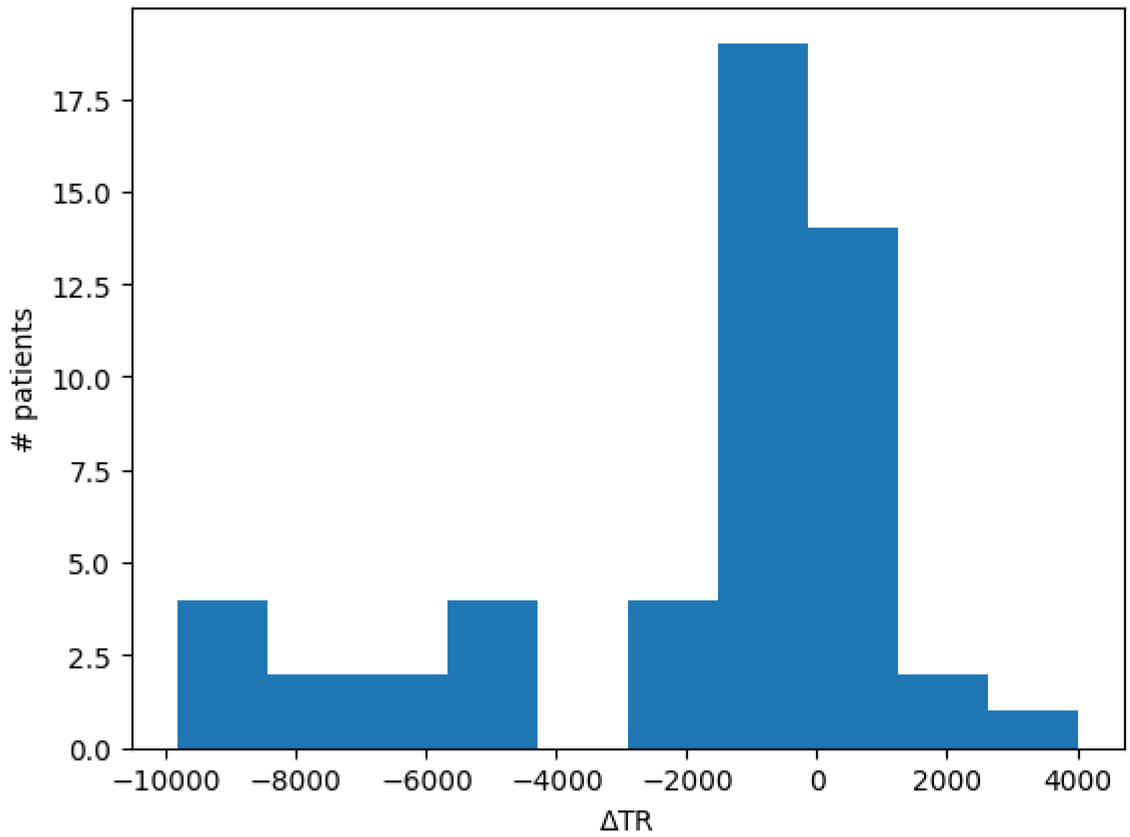

By reasonably grouping ΔTR while ensuring that each bin will have at least 10 patients to estimate CCC with sufficient statistical power (at least for excellent features), we considered three ΔTR intervals: -9840<=ΔTR<-4000 (n.10), -4000<=ΔTR<0 (n.16) and finally ΔTR≥0 (n.26)

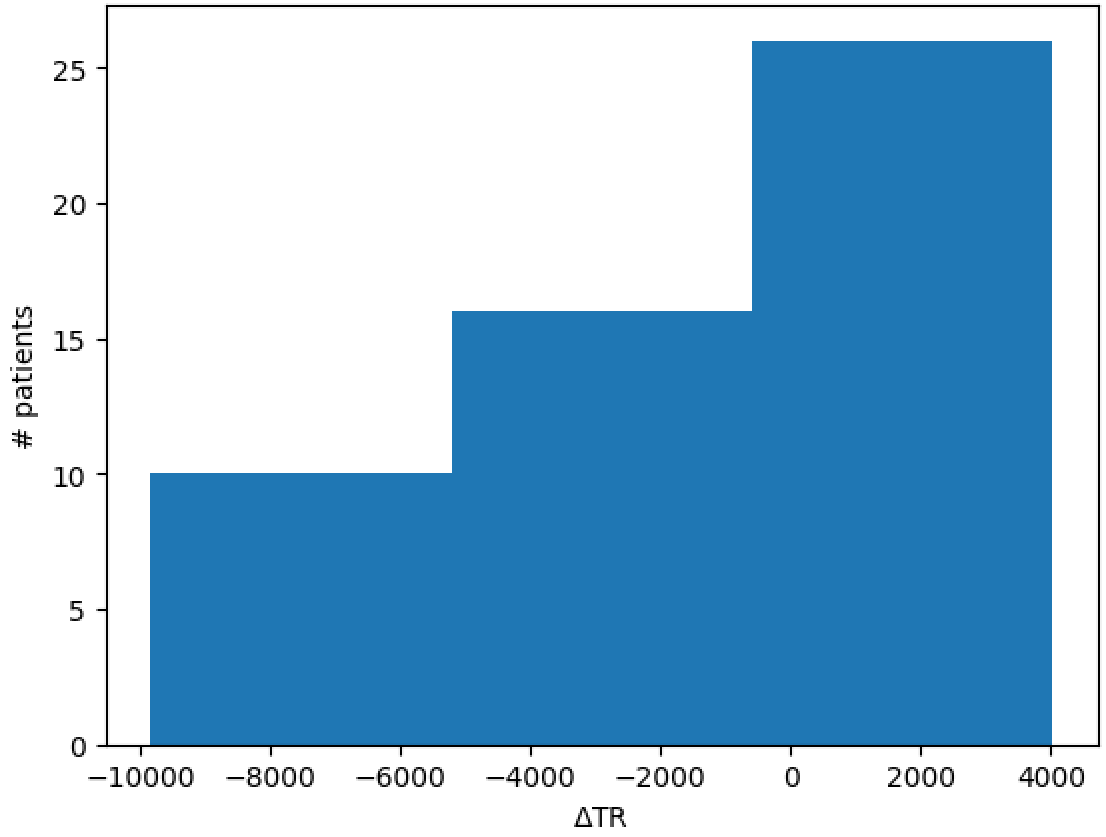

For each radiomics feature, we calculated the CCC within each ΔTR bin and attempted to fit a linear regression model. Given the multiplicity effect, conducting 1911 independent significance tests at α=0.05 is expected to yield approximately 95.5 false positives (1911 × 0.05 = 95.55). In fact, overall, there were 62 p-values < 0.05. To address this, we applied the Benjamini-Hochberg correction. After correction, no features remained statistically significant. The table below presents the excellent features that were significant before correction (n=9).

**Feature**	**Slope**	**P-value**	**Corr. P-value**
original_ngtdm_Busyness	-0.000005	0.024117	0.897895
log-sigma-2-0-mm-3D_glszm_GrayLevelNonUniformity	0.000015	0.010437	0.897895
wavelet-LLH_glcm_Idm	0.000017	0.002880	0.897895
wavelet-LLH_ngtdm_Strength	-0.000016	0.017419	0.897895
wavelet-HLL_ngtdm_Busyness	-0.000007	0.009482	0.897895
wavelet-HLL_gldm_SmallDependenceEmphasis	-0.000015	0.022130	0.897895
square_gldm_GrayLevelNonUniformity	-0.000013	0.018960	0.897895
gradient_glszm_GrayLevelNonUniformity	0.000020	0.001661	0.897895
gradient_gldm_DependenceEntropy	0.000007	0.001723	0.897895

Section S2: Details on the brain tissue- and filter-specific stability

Gm and csf features extracted with the best processing configuration were characterized by low excellent-level stability (6%) whereas 22.5% and 15.7% of features, respectively, presented at least good stability. On the other hand, wm showed a high stability profile with 11.7% of features exhibiting excellent and 33.1% showing at least good stability. For features extracted from tissue combinations other than gwm, 7.7% of brain features demonstrated excellent stability.
Apart from gwm features, this increase in excellent-level feature stability was also observed with other tissue sub-categories—brain, wm, gm, and, csf—where, the best pre-processing method, when coupled with Combat, led to an overall improvement of 33.3%, 46.7%, 51.9%, and 57.7%, respectively.

Filters
Unfiltered or original features comprised a mere 3.9% of stable excellent features, while filtered features accounted for the remaining 96.1% (wavelet, 37.3%; gradient, 19.6%; LBP, 18.1%; LoG, 14.7%), with a prevalence of texture features (88.7%). Of these, texture features constituted the highest fraction—88.7%, specifically GLRLM, and GLDM, both constituting nearly 21% followed by NGTDM (19.1%). In terms of the influence of filtering strategies, wavelet-filtered features constituted the highest percentage of stable excellent features (37.3%), followed by the gradient (19.6%), LBP (18.1%), and finally LoG filters (14.7%). Features based on exponential filters constituted the smallest fraction, accounting for less than 0.5%. Conversely, while considering excellent first-order features

separately, LoG (30.4%) and gradient (26.1%) filters showed higher stability than wavelet (17.4%) filters.
